# Asymmetric Transcription Factor Partitioning During Yeast Cell Division Requires the FACT Chromatin Remodeler and Cell Cycle Progression

**DOI:** 10.1534/genetics.120.303439

**Published:** 2020-09-02

**Authors:** Eva Herrero, Sonia Stinus, Eleanor Bellows, Lisa K. Berry, Henry Wood, Peter H. Thorpe

**Affiliations:** * Department of Plant Sciences, University of Cambridge, CB2 3EA, United Kingdom; † Laboratoire de Biologie Cellulaire et Moléculaire du Contrôle la Prolifération (LBCMCP), Centre de Biologie Intégrative (CBI), Centre National de la Recherche Scientifique (CNRS), Université de Toulouse, UT3, 31062, France; ‡ School of Biosciences, The University of Nottingham, Sutton Bonington, LE12 5RD, United Kingdom; § School of Biological and Chemical Sciences, Queen Mary, University of London, E1 4NS, United Kingdom

**Keywords:** Ace2, asymmetry, cell cycle

## Abstract

The polarized partitioning of proteins in cells underlies asymmetric cell division, which is an important driver of development and cellular diversity. The budding yeast *Saccharomyces cerevisiae* divides asymmetrically, like many other cells, to generate two distinct progeny cells. A well-known example of an asymmetric protein is the transcription factor Ace2, which localizes specifically to the daughter nucleus, where it drives a daughter-specific transcriptional network. We screened a collection of essential genes to analyze the effects of core cellular processes in asymmetric cell division based on Ace2 localization. This screen identified mutations that affect progression through the cell cycle, suggesting that cell cycle delay is sufficient to disrupt Ace2 asymmetry. To test this model, we blocked cells from progressing through mitosis and found that prolonged metaphase delay is sufficient to disrupt Ace2 asymmetry after release, and that Ace2 asymmetry is restored after cytokinesis. We also demonstrate that members of the evolutionarily conserved facilitates chromatin transcription (FACT) chromatin-reorganizing complex are required for both asymmetric and cell cycle-regulated localization of Ace2, and for localization of the RAM network components.

## Abstract (precis)

Most cell divisions are asymmetric with some cellular components distributed preferentially to one of the two nascent daughter cells. These asymmetries are typically important for the developmental fate of the resulting daughter cells. Herrero *et al.* describe .....

ASYMMETRIC cell division is a universal feature of life and provides a key mechanism to create different cell types. It is particularly important in adult stem cells, where asymmetric cell division maintains a stem cell pool, while generating progenitor cells to repair or replace damaged tissue (Neumuller and Knoblich 2009). Asymmetric cell division utilizes the polarity axis of the cell to align the mitotic spindle such that the plane of cell division is perpendicular to the axis of cell polarity. In this way, polarized proteins are partitioned differentially into the two daughter cells, potentially altering their fates (Neumuller and Knoblich 2009). Hence, identifying the mechanisms driving the distribution of the asymmetric proteins via cell polarity is fundamentally important to understand stem cell function and cell fate determination.

There are a number of mechanisms by which proteins can be polarized, ranging from external polarity cues to intrinsic positive and negative feedback that can establish polarity determinants (Johnson *et al.* 2011; Thompson 2013). The budding yeast *Saccharomyces cerevisiae* divides asymmetrically during every cell division. The mother cell divides by producing a small protrusion, known as the bud, that grows to produce a new daughter cell. The asymmetrical distribution of proteins between the mother and the daughter cell leads to a range of divergent phenotypes between these two cells. For example, mother cells progressively age with each cell division, senescing after ∼30 divisions. In contrast, this replicative ageing process is reset in the daughters, which are then themselves able to divide ∼30 times as new mothers (Denoth Lippuner *et al.* 2014). Proteins that are not intrinsically polarized can become so during cell division by selective protein localization to either the mother or the daughter cell (Yang *et al.* 2015). This process is typically driven by the activity of upstream, polarized proteins.

One such protein in *S. cerevisiae* is the transcription factor Ace2, which is restricted to the daughter cell nucleus in late anaphase. Ace2 regulates genes that are important for daughter cell (bud) specification, especially for the separation of the daughter cell from the mother cell and G1 delay (Dohrmann *et al.* 1992; Bidlingmaier *et al.* 2001; Colman-Lerner *et al.* 2001; Laabs *et al.* 2003; Bourens *et al.* 2008; Di Talia *et al.* 2009). Budding yeast undergoes closed mitosis and the dividing nucleus is highly compartmentalized, allowing nuclear import/export to be different in mother and daughter compartments (Boettcher and Barral 2013). Ace2 asymmetric localization is generated by the action of kinases and phosphatases that regulate Ace2’s nuclear localization (Figure 1A). *ACE2* is part of the “CLB2 cluster” of genes that are expressed from early M phase (Spellman *et al.* 1998). During early mitosis, a nuclear localization signal (NLS) within Ace2 is blocked by mitotic cyclin-dependent kinase (CDK) phosphorylation, which causes Ace2 to accumulate symmetrically in the cytoplasm (Dohrmann *et al.* 1992). During mitotic exit, the Cdc14 phosphatase is released into the cytoplasm. Cdc14 removes CDK phosphorylation from the Ace2 NLS allowing Ace2 nuclear entry (Archambault *et al.* 2004; Mazanka *et al.* 2008; Sbia *et al.* 2008). Ace2 accumulates only weakly in both the nascent mother and daughter nuclei because it is actively exported out of the nucleus, due to a nuclear export signal (NES) sequence (Jensen *et al.* 2000; Bourens *et al.* 2008). The RAM (regulation of Ace2 activity and cellular morphogenesis) network kinase Cbk1 phosphorylates the Ace2 NES, blocking Ace2 nuclear export (Mazanka *et al.* 2008; Sbia *et al.* 2008; Brace *et al.* 2011) (Figure 1A). Although the components of the RAM network localize to the bud neck and daughter cortex during telophase, it is still unclear how the RAM-mediated Ace2 accumulation is restricted to the daughter nucleus (Weiss 2012).

**Figure 1:**
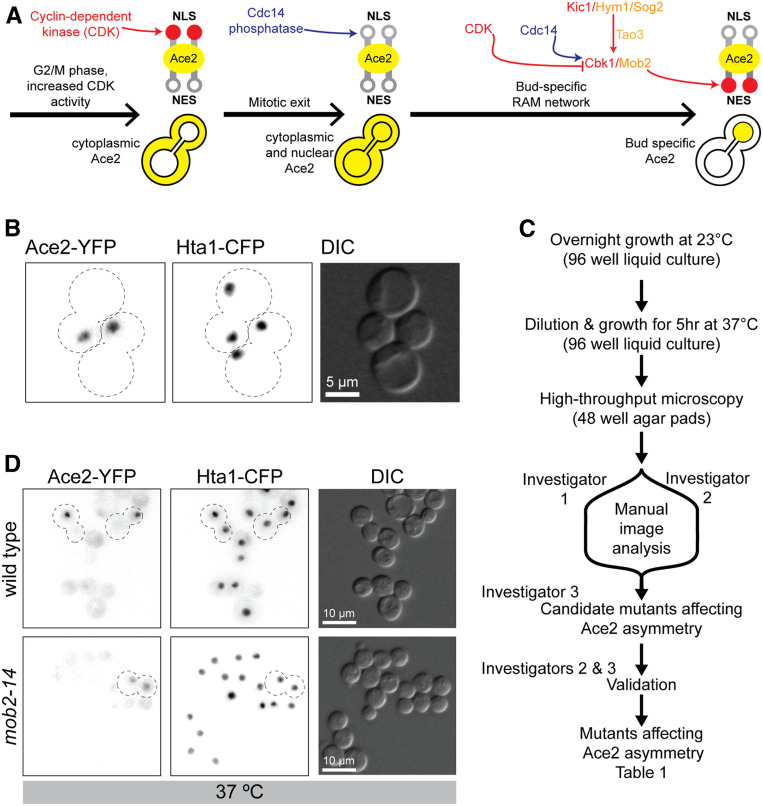
Reverse genetic screen to identify essential genes affecting Ace2 asymmetric localization. (A) Sequential phosphorylation and dephosphorylation controls Ace2 asymmetric localization. Kinase activity is shown in red (CDK, Kic1, and Cbk1), phosphatase activity is shown in blue (Cdc14), and nonkinase members of the RAM network are shown in orange (Hym1, Sog2, Tao3, and Mob2). Ace2 is illustrated in yellow. Both the NLS and NES of Ace2 can be deactivated by phosphorylation (shown in red). (B) Representative fluorescence image of two telophase cells of the W303 *HTA1-CFP* and *ACE2-YFP* strains crossed with the temperature-sensitive collection, PT31-75D. (C) Fluorescence microscopy screen and image analysis workflow. (D) Fluorescence imaging of wild-type and *mob2-14* at the restrictive (37°) temperature. CDK, cyclin-dependent kinase; CFP, cyan fluorescent protein; NES, nuclear export signal; NLS, nuclear localization signal; YFP, yellow fluorescent protein.

The RAM network is one of the yeast Mst/*hippo* or Ndr/LATS signaling systems that are present in most eukaryotic organisms. Mutations of any of the RAM network members results in cell separation defects and loss of asymmetric Ace2 localization (Bidlingmaier *et al.* 2001; Nelson *et al.* 2003). Cbk1 is the only “Ndr” kinase in yeast and requires its coactivator Mob2 to function. Cbk1-Mob2 interaction is constitutive throughout the cell cycle. The Cbk1-Mob2 complex also accumulates in the daughter cell nucleus in an Ace2-dependent manner (Colman-Lerner *et al.* 2001; Weiss *et al.* 2002). Cbk1 is activated, in part, by the “*hippo*” kinase Kic1, which works with its coactivator Hym1 (Nelson *et al.* 2003; Brace *et al.* 2011) and the other key components of the RAM network Sog2 and Tao3 (Jansen *et al.* 2006) (Figure 1A). In addition to Kic1 phosphorylation, Cdc14 release is also required to remove the inhibitory CDK phosphorylation from Cbk1, which in turn allows Cbk1 to interact with Ace2 (Figure 1A). Hence, Cbk1 phosphorylation of Ace2-NES is restricted to mitotic exit (Brace *et al.* 2011). However, it is the asymmetric distribution of Cbk1 that is responsible for Ace2’s asymmetry. Therefore, we wanted to ask which proteins regulate Ace2 asymmetry. In early G1, Cbk1 keeps Ace2 phosphorylated (Mazanka and Weiss 2010). Eventually during G1 progression, Ace2 is dephosphorylated and exported into the cytoplasm where a sequestration mechanism, involving either Cdk1 or Pho85, prevents Ace2 from reentering the nucleus (Mazanka and Weiss 2010).

A number of cell polarity screens have been performed in yeast. Initially, forward genetic screens were used to identify many of the important genes required for cell polarity, for example, *CDC42* (Adams *et al.* 1990). The creation of arrays of deletions of nonessential genes and overexpression plasmids has enabled the use of reverse genetic approaches to study the cell polarity (Haarer *et al.* 2007, 2011; Sopko *et al.* 2007; Zou *et al.* 2009); see Styles *et al.* (2013) for a review. The systematic fluorescence imaging of the GFP collection identified hundreds of proteins that localize to sites of polarization such as the bud neck, bud tip, or actin cytoskeleton (Huh *et al.* 2003). These screens have been highly informative for understanding the regulation of cell polarity and asymmetric cell division. However, systematic loss-of-function studies with essential genes had not been possible until the creation of collections of hypomorphic and temperature-sensitive alleles (Breslow *et al.* 2008; Li *et al.* 2011).

Here, we used Ace2 localization as a reporter to test the contribution of essential cellular processes to the maintenance of cell polarity and asymmetry. We performed a fluorescence microscopy screen to assay the localization of Ace2 in an array of temperature-sensitive mutants of essential genes. Many of the mutants that affected Ace2 asymmetry are involved in mitotic cell division, and we found that mitotic delay is sufficient to decrease Ace2 asymmetry. In addition, we identified the facilitates chromatin transcription (FACT) complex as essential to maintain Ace2 asymmetry, its cell cycle-regulated nuclear localization, and the localization of the upstream Ndr kinase Cbk1.

## Materials and Methods

### Strains

A full list of strains is included in Supplemental Material, Table S1 and a full list of temperature-sensitive alleles tested in the screen is included in Table S2. Yeast medium and growth was performed using standard methods (Sherman 2002). We constructed a strain (PT31-75D) that includes *HTA1-CFP*::*HYG* and *ACE2-YFP*::*NAT* in addition to the haploid-specific marker *lyp1*∆::*STE3pr-LEU2*. We crossed this *MAT*α strain with the 1334 members of the *MAT***a** temperature-sensitive collection *geneX-ts*::*KANMX* (Li *et al.* 2011) using the synthetic genetic array (SGA) technology (Tong *et al.* 2001) and employing a ROTOR pinning robot (Singer Instruments) to copy the cells on the different selection media. Diploids were selected on YPD with geneticin (G418) and nourseothricin (NAT), and then sporulated in sporulation media. The resulting spores were selected on synthetic medium lacking leucine and supplemented with 50 µg/ml thialysine. These cells were copied sequentially onto media supplemented with G418, NAT, and hygromycin (HYG) to select for fluorescently tagged and temperature-sensitive alleles.

To generate an auxin-inducible degron (AID) of Spt16, we created *SPT16-AID*::*HYG ura3-1*::*ADH1pAFB2*, the plasmid pHT453 was linearized with StuI and integrated into the genome of strains containing the appropriate fluorescent reporters. Then, the *AID-6XFLAG*::*HYG* cassette was amplified from plasmid pX58 (see primers Table S1) and integrated at the endogenous *SPT16* gene. Transformed strains were confirmed by PCR and Sanger sequencing. Cell viability was assayed by spotting cells onto plates supplemented with 500 μM auxin.

### Microscopy

For the screen, cells were prepared for imaging by growth in ∼250 µl of liquid synthetic media supplemented with adenine (100 mg/liter, +Ade) in 96-well plates overnight at 23°. These cultures were then diluted (1:10) and grown at 37° for 5 hr. These yeast cultures were imaged on agar pads (Werner *et al.* 2009) using a ×63, 1.4 NA oil immersion objective lens (Zeiss [Carl Zeiss], Thornwood, NY). Fluorophores were excited with light-emitting diode (LED)-based illumination at 445 nm for cyan fluorescent protein (CFP) and 505 nm for yellow fluorescent protein (YFP) using appropriate filter sets (47HE for CFP and 46HE for YFP; Zeiss). Fluorescence images were acquired on a charge-coupled device (CCD) camera (Orca ERII, Hamamatsu Photonics K.K.) with exposure times of 10 msec for CFP and 150 msec for YFP. The CCD pixels were binned 2 × 2 for an improved signal-to-noise ratio.

For other microscopy, cells were grown overnight in the appropriate synthetic media (+Ade). On the day of the experiment, cells were diluted to OD_600_ = 0.3. For experiments using temperature-sensitive strains, cells were grown for 5 hr at a permissive or restrictive temperature. For auxin-dependent Spt16-AID depletion experiments, log-phase cultures at OD_600_ = 0.3 were grown for 1 hr before adding 500 µM auxin (from 100 mM stock in 100% ethanol) or only ethanol. Then, cells were grown for 5 hr before imaging. For *CRM1-OX* experiments, cells were grown in synthetic media with 2% raffinose and 0.1% glucose. An additional 2% galactose was added to induce *CRM1-OX* from the GAL1 promoter.

For metaphase arrest experiments, *MET3pCDC20* strains were grown overnight in synthetic media lacking methionine (−Met). The day of the experiment, cultures were diluted to OD_600_ = 0.3 and grown for 1 hr. Then, cultures were spun down and resuspended in synthetic media with 2 mM methionine (+Met) and grown for the indicated time. To release from the arrest, cultures were washed twice with distilled water and resuspended in –Met. Then, cells were grown and imaged every 30 min to follow mitotic progression.

For fluorescence imaging, cells were mounted on microscope slides with 0.7% low melting point agarose in the appropriate synthetic media +Ade, and covered with 0.17-mm glass coverslips. Cells were imaged with a ×63, 1.4 NA oil immersion objective lens (Zeiss). YFP and CFP fluorophores were excited as explained above. Additionally, GFP and red fluorescent protein (RFP) were excited with LED-based illumination at 470 nm for GFP and 590 nm for RFP using appropriate filter sets (38HE for CFP and 60HE for RFP; Zeiss). Images were captured either with a CCD camera (see above) or a complementary metal-oxide-semiconductor camera (Flash 4.0 lte, Hamamatsu Photonics K.K.).

### Image analysis

For the screen, images were scored manually using Volocity imaging software (Perkin-Elmer [Perkin Elmer-Cetus], Norwalk, CT). Two investigators scored the microscope images independently by visual assessment according to the criteria listed in Table S2, principally to determine whether Ace2 was localized asymmetrically or symmetrically in late mitosis (Figure 1D). A third investigator independently resolved discrepancies in the resulting scores to produce a preliminary list of hits. We then retested these preliminary hits and other mutant strains whose genes were functionally or physically associated with the preliminary hits, but were not scored or did not register as a hit in the original screen.

**Figure 2:**
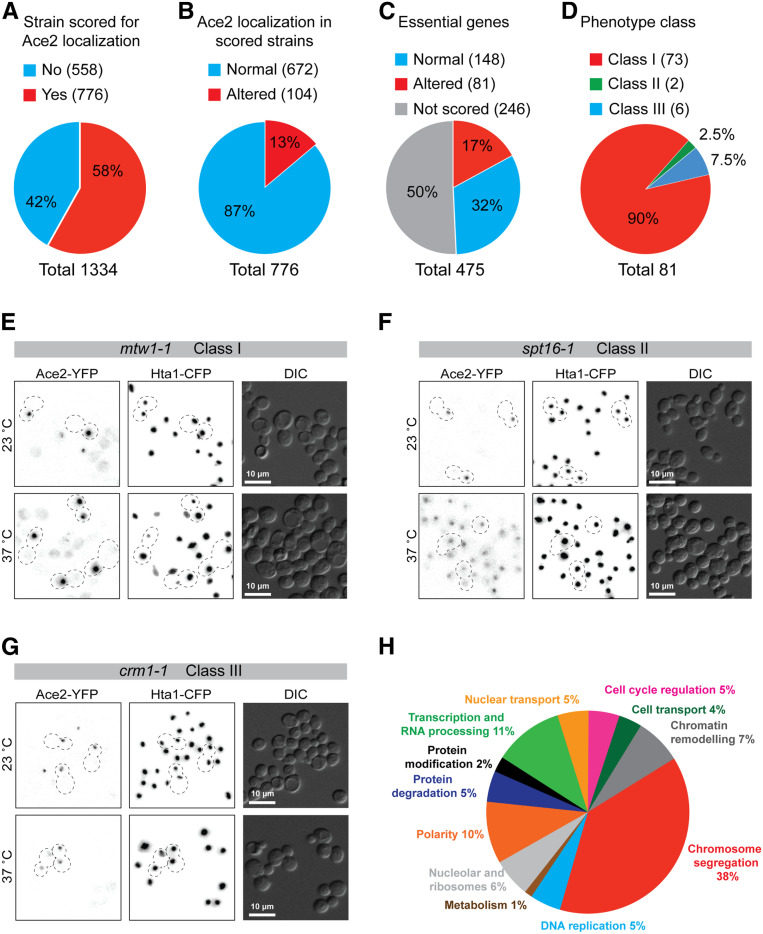
Results from Ace2-YFP reverse genetic screen. (A–D) Summary of screen results: (A) proportion of total strains scored for Ace2-YFP localization, (B) characterization of Ace2 localization, (C) classification of essential genes according to Ace2 localization phenotype, and (D) phenotypic classification of altered Ace2 localization. (E) Fluorescence microscopy imaging of the class I mutant *mtw1-1* at 23° and 37°. (F) Fluorescence microscopy imaging of class II mutant *spt16-1* at 23° and 37°. (G) Fluorescence microscopy imaging of class III mutant *crm1-1* at 23° and 37°. (H) Annotated cellular function of mutants found to alter Ace2-YFP asymmetric localization. See Table 1. CFP, cyan fluorescent protein; YFP, yellow fluorescent protein.

**Figure 3:**
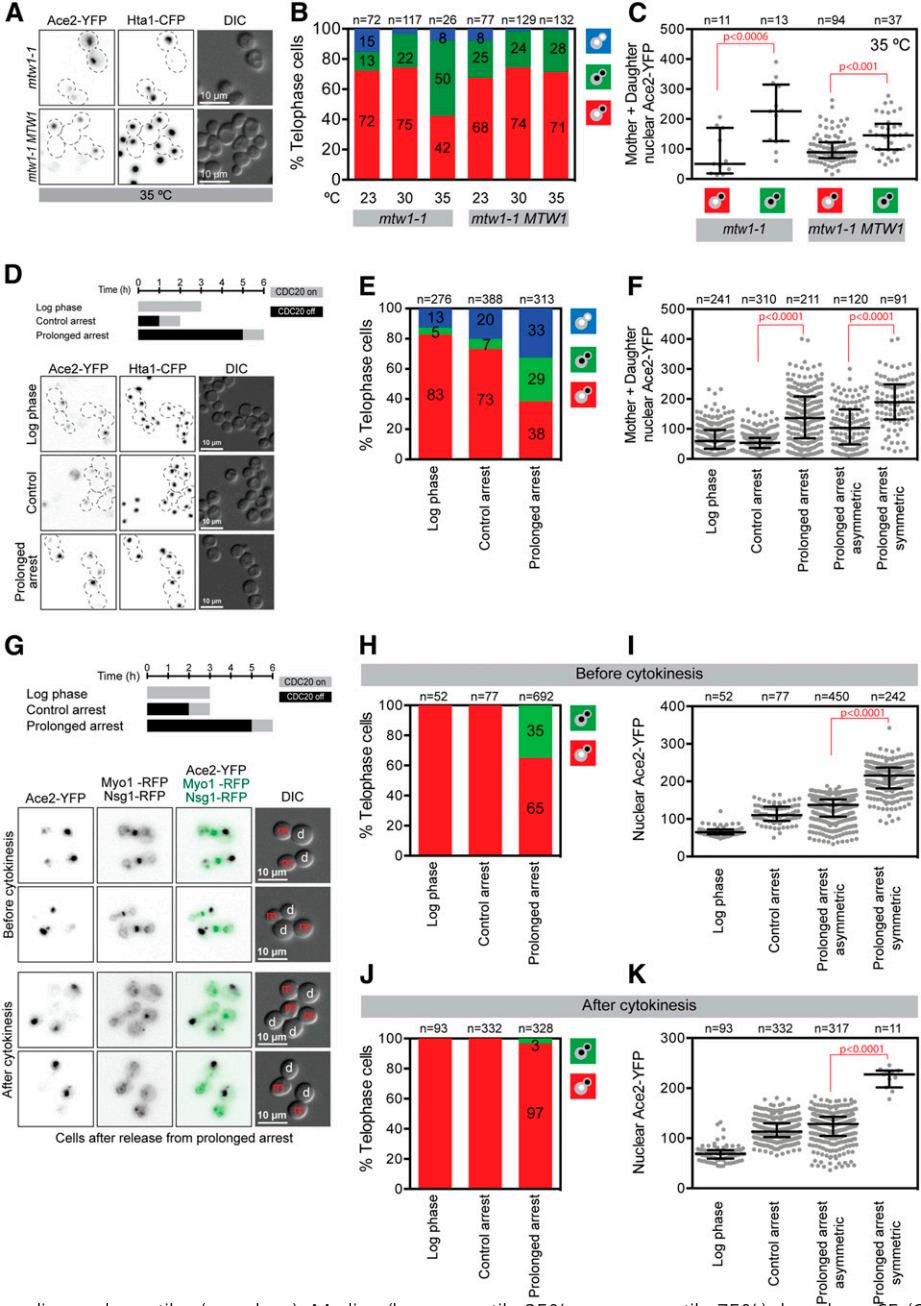
Slow cell cycle progression decreases Ace2-YFP asymmetric localization. (A) Representative fluorescence microscopy images of *mtw1-1* and *mtw1-1 MTW1* growing for 5 hr at 35°. (B) Quantitation of Ace2-YFP asymmetry of *mtw1-1* and *mtw1-1 MTW1* cells at three different temperatures. (C) Mother + daughter nuclear Ace2-YFP fluorescence intensity. The plot shows the median and quartiles (error bars). Median (lower quartile 25%, upper quartile 75%): *mtw1-1* asymmetric 50 (19, 171), *mtw1-1* symmetric 226 (126, 315), *mtw1-1 MTW1* asymmetric 89 (70, 123), and *mtw1-1 MTW1* symmetric145 (98, 184). (D) Prolonged metaphase arrest experimental setup and representative fluorescence microscopy images. Control arrest and prolonged arrest cells were imaged from 30 min after release from CDC20 arrest. (E) Quantitation of Ace2-YFP asymmetry after prolonged arrest. (F) Mother + daughter nuclear Ace2-YFP fluorescence intensity. The plot shows the median and quartiles (error bars). Median (lower quartile 25%, upper quartile 75%): log phase 60 (34, 97), control arrest 54 (36, 70), prolonged arrest all telophase cells 137 (68, 208), prolonged arrest asymmetric 103 (48, 165), and prolonged arrest symmetric 190 (132, 249). (G) Prolonged metaphase arrest experimental setup and representative fluorescence microscopy imaging of cells released from prolonged metaphase arrest before and after cytokinesis. See additional images in Figure S6. Control arrest and prolonged arrest cells were imaged from 30 min after release from CDC20 arrest. Cells were assigned to “before cytokinesis” or “after cytokinesis” categories as determined by the presence or absence of Myo1-RFP bud neck signal, respectively. Mother (m) and daughter (d) cells are indicated in the DIC panel. Note than due to the prolonged metaphase, daughter cells are larger than mother cells. (H) Quantitation of Ace2-YFP asymmetry of cells before cytokinesis from experiment in (G). (I) Mother + daughter nuclear Ace2-YFP fluorescence intensity of cells before cytokinesis from experiment in (G). The plot shows the median and quartiles (error bars). Median (lower quartile 25%, upper quartile 75%): log phase 65 (60, 72), control arrest 110 (95, 133), prolonged arrest asymmetric 138 (106, 152), and prolonged arrest symmetric 210 (181, 237). (J) Quantitation of Ace2-YFP asymmetry of cells after cytokinesis from experiment in panel G. (I) Mother + daughter nuclear Ace2-YFP fluorescence intensity of cells after cytokinesis from experiment in (G). The plot shows the median and quartiles (error bars). Median (lower quartile 25%, upper quartile 75%): log phase 69 (59, 76), control arrest 113 (102, 130), prolonged arrest asymmetric 128 (105, 143), and prolonged arrest symmetric 227 (201, 235). *P*-values in panels C, F, I, and K calculated with Kruskal–Wallis test. CFP, cyan fluorescent protein; RFP, red fluorescent protein; YFP, yellow fluorescent protein.

For quantitation of Ace2-YFP, we used a custom-made protocol in Volocity imaging software. Hta1-CFP fluorescence intensity was used to automatically segment yeast nuclei. A background region was selected by subtracting a 2× dilation from a 4× dilation of the yeast nuclei. For every object (nuclei and background), both CFP and YFP mean fluorescence was measured. Background mean intensity was subtracted from nuclei mean intensity to calculate the corrected nuclear intensity. Telophase cells were manually selected from the images. The corrected Ace2-YFP nuclear intensity of S-phase cells was used as a threshold to define nuclei without Ace2-YFP. The presence or absence of Ace2-YFP in either one or both nascent mother or daughter nuclei was used to assign telophase cells to the categories indicated in Figure 2 and Figure 3. Cumulative fluorescence intensity between mother and daughter cells of nuclear Ace2-YFP was calculated by the sum of the corrected Ace2-YFP of both nascent nuclei of telophase cells. For the experiment in Figure 3, H–K, automatic nuclei segmentation was achieved by using Ace2-YFP intensity; thus, cells without Ace2-YFP either in mother or daughter nuclei are not included in the analysis. Bud neck-localized Myo1-RFP was automatically segmented using RFP fluorescent intensity. The images shown in Figure 5, A, B, and D were deconvolved for increased clarity. We used the deconvolution algorithm in Volocity software (Perkin-Elmer) using a simulated point-spread function.

### Western blot analysis

Whole-cell extracts were prepared as previously described (Olafsson and Thorpe 2015). First, 10 μl of protein extracts were loaded in a 7.5% acrylamide gel (Bio-Rad, Hercules, CA). Proteins were transferred onto a 0.45-μm supported nitrocellulose membrane (Bio-Rad). Membrane blocking and antibody incubation were performed using western blocking reagent (Roche). Anti-FLAG (F7425; Sigma [Sigma Chemical], St. Louis, MO) and anti-GFP antibodies (11814460001; Roche) were used at 1:1000 dilutions. Anti-Pgk1 antibody (459250; Invitrogen, Carlsbad, CA) was used at 1:10000. HRP-conjugated anti-rabbit IgG (A0545; Sigma) and anti-mouse IgG (ab97265; Abcam) were used at 1:100000 and 1:30000 dilution, respectively. Membranes were incubated for 1 min with Lumi-Light western blotting substrate (Roche) before film exposure.

### Data availability

Strains and plasmids are available upon request. The authors affirm that all data necessary for confirming the conclusions of the article are present within the article, figures, tables, and supplementary files. Supplemental material available at figshare: https://doi.org/10.25386/genetics.1290118.

## Results

### Identification of essential genes involved in Ace2 asymmetry

To assess which essential genes are required for Ace2 asymmetry, we made use of a collection of temperature-sensitive mutants of essential genes. This set consists of 1334 isolates of 787 temperature-sensitive alleles in 497 genes, which covers about one-half of the essential genes in yeast; each allele is linked to a *KANMX* selectable marker (in the BY4741 background, (Li *et al.* 2011). To assay for Ace2 asymmetry, we created a strain (PT31-75D, W303 background) that encodes Ace2 linked to YFP (Ace2-YFP) together with a histone H2 peptide (Hta1) fused to CFP (Hta1-CFP) (Figure 1B). Both genes are tagged at their endogenous loci in the genome and remain under the control of their native promoters. This strain shows the characteristic asymmetric distribution of Ace2 in late mitosis (Figure 1, A and B). Since these two fluorescently marked alleles are linked to selectable markers (NAT resistance for *ACE2-YFP* and hygromycin resistance for *HTA1-CFP*), we were able to cross this strain with the temperature-sensitive collection of mutants using the SGA methodology (Tong *et al.* 2001). This method allowed us to create haploid strains containing the two fluorescent markers in addition to the temperature-sensitive allele in a hybrid W303-BY4741 background. These strains were then assayed for Ace2 asymmetry using fluorescence microscopy after 5 hr of incubation at their restrictive temperature (37°). Two independent investigators visually scored the resulting microscope images by visual assessment of whether Ace2 localized asymmetrically and or symmetrically in late mitosis; discrepancies in the resulting scores were resolved by a third investigator (Figure 1C). We included a number of wild-type controls to ensure that Ace2 asymmetric localization was unaffected by the method (Figure 1D). Additionally, we used a temperature-sensitive allele of *MOB2* (*mob2-14*) as a positive control (Figure 1D).

Of the 1334 temperature-sensitive strains examined, we were able to score 743 (56%) for Ace2 distribution. There were three main reasons why we could not score the remaining 44%: (1) some cells did not grow after the SGA procedure, (2) some strains did not produce telophase cells after incubation at the restrictive temperature, and (3) some images were of insufficient quality to score Ace2 distribution. Most of the resulting mutant alleles that were scored as affecting Ace2 asymmetry were then retested individually to remove false positives. To reduce false negatives, we retested mutant strains whose genes were functionally or physically associated with the preliminary hits, but that we were unable to score in the original screen (33 strains) or were not a preliminary hit (49 strains). This increased our total number of scored strains to 776 (58% of the total temperature-sensitive alleles, Figure 2A). A full list of all the strains and alleles screened plus their Ace2 phenotype is listed in Table S2. We found that 104 strains showed evidence of asymmetry breaking at the restrictive temperature (Figure 2B and Table S2), which comprised mutations in 81 genes (Figure 2C and Table 1). We divided the 81 genes into three phenotypic classes according to the localization of Ace2 (Figure 2D). Class I was the largest phenotypic category (90% of mutant genes) and represented a partial phenotype where some cells had wild-type Ace2 asymmetric localization, while other cells showed symmetric Ace2 localization in mother and daughter cells (see Figure 2E for example). Two class II mutants showed a complete loss of asymmetric localization of Ace2 and Ace2 was present in all cell nuclei regardless of cell cycle stage (Figure 2F). Finally, six class III mutants showed symmetric localization of Ace2 that was restricted to cells in late mitosis (Figure 2G). We examined collectively the cellular role of the genes whose mutants affected Ace2 localization (Table 1). A significantly large proportion of them were involved in chromosome segregation (38% of the genes, Fisher’s exact test *P* = 2 × 10^−10^, Figure 2H). Other represented functions were polarity, chromatin remodeling, transcription and RNA processing, and cell cycle regulation (Figure 2H and Table 1).

**Table 1:** List of genes found to affect Ace2-YFP asymmetry

Protein	TS mutant	Molecular function
**Class I**		
Apc5	*apc5-CA-Paps*	Cell cycle regulation*^a^*
Cdc27	*cdc27-2*	Cell cycle regulation*^a^*
Cdc28	*cdc28-13*	Cell cycle regulation*^a^*
Cdc20	*cdc20-2*, *cdc20-3*	Cell cycle regulation*^a^*
Bet2	*bet2-1*	Cell transport
Sed5	*sed5-1*	Cell transport
Sft1	*sft1-15*	Cell transport
Abf1	*abf1-102*	Chromatin remodeling
Rsc8	*rsc8-ts16*	Chromatin remodeling
Swd2	*swd2-1*	Chromatin remodeling
Tel2	*tel2-15*	Chromatin remodeling
Ndc10	*ndc10-1*	Chromosome segregation*^a^*
Cdc14	*cdc14-8*	Chromosome segregation*^a^*
Cdc31	*cdc31-2*	Chromosome segregation*^a^*
Cse4	*cse4-1*	Chromosome segregation*^a^*
Dad2	*dad2-9*	Chromosome segregation*^a^*
Dam1	*dam1-1*, *dam1-19*	Chromosome segregation*^a^*
Duo1	*duo1-2*	Chromosome segregation*^a^*
Eco1	*eco1-1*	Chromosome segregation*^a^*
Esp1	*esp1-1*	Chromosome segregation*^a^*
Ipl1	*ipl1-2*	Chromosome segregation*^a^*
Mif2	*mif2-3*	Chromosome segregation*^a^*
Mps1	*mps1-1*	Chromosome segregation*^a^*
Mtw1	*mtw1-ts*	Chromosome segregation*^a^*
Nbp1	*nbp1-1*	Chromosome segregation
Nnf1	*nnf1-17*, *nnf1-48*, *nnf1-77*	Chromosome segregation*^a^*
Nsl1	*nsl1-5*, *nsl1-6*	Chromosome segregation*^a^*
Nuf2	*nuf2-61*	Chromosome segregation*^a^*
Pds1	*pds1-128*	Chromosome segregation*^a^*
Sgt1	*sgt1-3*, *sgt1-5*	Chromosome segregation
Sli15	*sli15-3*	Chromosome segregation
Smc1	*scm1-1*	Chromosome segregation*^a^*
Smc3	*scm3-42*	Chromosome segregation*^a^*
Spc110	*spc110-220*	Chromosome segregation*^a^*
Spc24	*spc24 4-2*	Chromosome segregation*^a^*
Spc25	*spc25-1*	Chromosome segregation*^a^*
Spc29	*spc29-20*	Chromosome segregation
Spc34	*spc34 41-1*	Chromosome segregation*^a^*
Stu1	*stu1-12*, *stu1-5*, *stu1-6*, *stu1-7*	Chromosome segregation*^a^*
Stu2	*stu2-11*, *stu2-13*	Chromosome segregation*^a^*
Dbf4	*dbf4-2*, *dbf4-3*, *dbf4-ts*	Chromosome segregation*^a^*
Smt3	*smt3-42*	Chromosome segregation*^a^*
Cdc6	*cdc6-1*	DNA replication*^a^*
Cdc7	*cdc7-4*	DNA replication*^a^*
Cdc21	*cdc21-ts*	DNA replication
Psf1	*psf1-1*	DNA replication
Kre5	*kre5-ts2*	Metabolism
Krr1	*krr1-18*	Nucleolar and ribosome
Nog2	*nog2-1*	Nucleolar and ribosome
Nop2	*nop2-5*, *nop2-9*	Nucleolar and ribosome
Nop7	*nop7-1*	Nucleolar and ribosome
Rrp5	*rrp5-delta6*	Nucleolar and ribosome
Arp3	*arp3-G302Y*	Polarity
Cdc24	*cdc24-5*	Polarity*^a^*
Cdc43	*cdc43-2*	Polarity
Exo70	*exo70-20/37*	Polarity
Pan1	*pan1-4*	Polarity*^a^*
Sec23	*sec23-1*	Polarity
Cdc34	*cdc34-1*	Protein degradation*^a^*
Met30	*met30-6*	Protein degradation
Pre2	*pre2-75*	Protein degradation
Rpt6	*rpt6-20*	Protein degradation
Gpi13	*gpi13-5*	Protein modification
Sec53	*sec53-6*	Protein modification
Cdc39	*cdc39-1*	Transcription and RNA
Hts1	*hst1-1*	Transcription and RNA
Prp19	*prp19-1*	Transcription and RNA
Prp2	*prp2-1*	Transcription and RNA
Rse1	*rse1-1*	Transcription and RNA
Slu7	*slu7-ts2*	Transcription and RNA
Ssl2	*ssl2-ts*	Transcription and RNA
Ssu72	*ssu72-2*	Transcription and RNA
Yhc1	*ych1-1*	Transcription and RNA
**Class II**		
Pob3	*pob3-7*, *pob3-L78R*	Chromatin remodeling
Spt16	*spt16-1*	Chromatin remodeling
**Class III**		
Crm1	*crm1-1*	Nuclear transport
Rna1	*rna1-1*	Nuclear transport
Srm1	*srm1-ts*	Nuclear transport
Yrb1	*yrb1-51*	Nuclear transport
Hym1	*hym1-15*	Polarity
Mob2	*mob2-14*, *mob2-19*, *mob2-22*, *mob2-28*, *mob2-36*, *mob2-38*	Polarity

The presence of the corresponding mutation was confirmed either by PCR genotyping or by sequencing in some strains of interest. We note that the identity of the rest of the strains in the screen has not been confirmed. TS, temperature-sensitive.

a Genes in gene ontology category “mitotic cell process.”

When validating the Ace2 phenotypes using complementation, we noticed that some colonies derived from the SGA procedure were temperature-sensitive despite containing a complementing gene for their mutant temperature-sensitive allele. Further investigation showed that the *HTA1-CFP* allele results in a temperature-sensitive phenotype specifically in the W303 genetic background, but not in the BY4741 background. We were able to show that the *SSD1* polymorphism (*ssd1-d*) present in W303 but not in BY4741, which encodes for a truncated Ssd1 protein with no function (Uesono *et al.* 1997), was responsible for the W303 temperature-sensitive phenotype of *HTA1-CFP* cells (Figure S2A). We also tested whether the *SSD1* allele could affect Ace2-YFP asymmetry. We found that *ssd1-d* strains grown at 37° had more telophase cells with no Ace2 in either mother or daughter nuclei. However, the percentage of symmetric cells (Ace2-YFP in both mother and daughter nuclei) was similar between cells with full-length Ssd1 (*SSD1-V)* and the truncated form (*ssd1-d)*. In the presence of a functional SSD1 (*SSD1-V*), the percentage of asymmetric cells was higher in W303/BY4741 hybrid cells than in pure W303 cells suggesting that other genetic loci affect Ace2 asymmetry (Figure S2B).

### Class III mutants are known regulators of Ace2 asymmetry

As previously reported, *crm1-1* cells showed strong symmetric localization of Ace2 in anaphase–telophase cells (Figure 2G) (Jensen *et al.* 2000; Bourens *et al.* 2008). We also found mutants of three additional components of the nuclear export machinery—Rna1, Yrb1, and Srm1—with a similar phenotype to *crm1-1* (Figure S3A and Table 1), as well as alleles encoding two components of the RAM signaling network that regulates Ace2 distribution, Hym1 and Mob2 (6 separate alleles) (Figure S3B and Table 1). The other members of this network, Kic1, Tao3, and Cbk1, are not represented in the temperature-sensitive collection. Therefore, class III mutants validated the ability of our screen to find regulators of Ace2 asymmetry.

### Class I mutants are enriched in mitotic cell cycle processes

Since 90% of our hits were class I mutants, we performed gene ontology enrichment analysis within class I genes (GOrilla) (Eden *et al.* 2009). We found a 2.6-fold enrichment for genes involved in “mitotic cell cycle process” when comparing the class I genes to all of the genes represented in the temperature-sensitive collection (enrichment *P*-value 3.8 × 10^−11^). Among these genes, 12 encoded structural kinetochore components such as Mtw1 (see below), spindle and spindle-pole body components such as Spc110, and mitotic regulators such as Cdc27, Ipl1, and Cdc20 (Table 1).

One of the class I mutants is *mtw1-1* (Figure 2E). Mtw1 is a structural protein of the kinetochore. To confirm that *mtw1-1* mutation specifically caused this phenotype, we imaged *mtw1-1* mutant cells and *mtw1-1 MTW1* cells (where the mutant allele was complemented with a wild-type copy of *MTW1* integrated at the *URA3* locus) at permissive (23° and 30°) and restrictive (35°) temperatures (see Figure 3A for 35° images), and quantified the loss-of-asymmetry phenotype. We used Ace2-YFP fluorescence signal as a surrogate for Ace2 concentration in both mother and daughter nuclei of telophase cells, and assigned cells to three different categories: cells with Ace2 only in one nucleus (asymmetric), cells with Ace2 in both mother and daughter nuclei (symmetric), and cells with no Ace2 in either nuclei (Figure 3B). We found that in the *mtw1-1* strain there was a reduction of cells with Ace2 in one nucleus (from 72 to 42%, Fisher’s exact test *P* = 0.007), and an increase in the number of cells with Ace2 in both mother and daughter nuclei (from 13 to 50%, Fisher’s exact test *P* = 0.002) at the restrictive temperature (35°) (Figure 3B). To measure the extent of loss-of-asymmetry of *mwt1*-1 symmetric cells, we calculated the asymmetry index (AI) by dividing the difference between the Ace2-YFP fluorescence intensities of the mother and daughter cells by the total fluorescence intensity of both nuclei. The AI values ranged from 1 (total asymmetry) to 0 (total symmetry) (Lengefeld *et al.* 2018). The AI of *mtw1-1* symmetric cells was 0.48 ± 0.28 (*n* = 13) (Table S3). The partial reduction of the AI in symmetric cells was consistent with a partial loss-of-asymmetry in *mtw1-1* cells at the restrictive temperature. The loss-of-asymmetry phenotype was rescued in *mtw1-1 MTW1* cells, thus confirming that *mtw1-1* mutation specifically caused the phenotype (Figure 3, A and B). We found that the cumulative Ace2-YFP fluorescence intensity of mother and daughter nuclei (mother + daughter nuclear Ace2-YFP) was significantly higher in *mtw1-1* symmetric cells than in asymmetric cells (Figure 3C).

Since the *mtw1-1* strain in our screen is a hybrid of W303 and BY4741 genetic backgrounds, we confirmed the partial Ace2 loss-of-asymmetry phenotype in a W303 isogenic strain (Figure S4). In the *mtw1-1* isogenic strain, the proportion of Ace2 asymmetric cells decreased from 69% at 23° to 29% at 35° (Fisher’s exact test *P* < 0.001), and the proportion of symmetric cells increased from 22% at 23° to 43% at 35° (Fisher’s exact test not significant; Figure S4, A and C). Introducing a wild-type *MTW1* copy complemented the *mtw1-1* phenotype, since in an *mtw1-1 MTW1* strain, the proportion of asymmetric cells was similar at 23° and 35° (Fisher’s exact test not significant, Figure S4A and S4C), indicating the complementation of *mtw1-1* with the wild-type MTW1 gene. In addition, in an *mtw1-1* W303 isogenic strain at 35°, cells with symmetric Ace2 had increased nuclear Ace2-YFP levels when compared with asymmetric cells (Figure S4B). In the *mtw1-1 MTW1* strain, asymmetric cells also have increased nuclear Ace2-YFP levels, albeit not statistically significant (Figure S4B).

### Metaphase arrest leads to partial loss-of-asymmetry

A common feature of all of these cell cycle-related mutants is that they will likely disrupt the normal progression of the cell cycle. Prolonged mitotic delay perturbs asymmetry of acentric DNA (Gehlen *et al.* 2011) and changes in nuclear shape can decrease the amount of Ace2 asymmetry (Boettcher *et al.* 2012). Thus, we hypothesized that in the class I mutants, Ace2 asymmetry is affected by delayed progression through mitosis. To test this hypothesis, we induced a defined mitotic delay and asked whether Ace2 distribution was affected in late anaphase. First, we used depletion of the Cdc20 protein to arrest cells prior to the completion of mitosis. A *CDC20* allele under the control of a *MET3* promoter is transcriptionally repressed by the addition of methionine, which arrests cells in metaphase (Uhlmann *et al.* 2000). We created a strain that includes the *MET3pr-CDC20* allele together with alleles encoding the tagged versions of Ace2 and Hta1. Cells were arrested in metaphase for either 1 hr (control arrest) or 5 hr (prolonged arrest). Cells were then released for 1 hr by transfer into methionine-deficient growth medium, thus allowing cells to enter anaphase and to progress to telophase, and then we imaged them (see Figure 3D for example). We quantitatively measured the level of asymmetry of Ace2 as described previously. A short (1 hr) metaphase arrest (control arrest) produced no detectable defect in Ace2 asymmetry when compared with log-phase cells (Figure 3E). In contrast, we found that prolonged arrest caused a significant reduction in the proportion of Ace2 asymmetric cells (Fisher’s exact test *P* = 9 × 10^−21^) and an increase in symmetric cells (Fisher’s exact test *P* = 7 × 10^−6^) when compared with the control arrest (Figure 3E). Moreover, we found that prolonged arrest caused increased cumulative levels of Ace2-YFP in telophase cells (mother + daughter nuclear Ace2-YFP). Specifically, symmetric cells had higher levels of nuclear Ace2 than asymmetric cells (Figure 3F). In contrast, G1 cell cycle delay using α-factor did not affect Ace2 asymmetry (Figure S5, A, B, and C). The increase in symmetric cells caused by prolonged arrest was similar to that in the *mtw1-1* mutant (Figure 3, B and E and Figure S4A). Both the *mtw1-1* mutant and prolonged arrest increased the levels of nuclear Ace2-YFP in symmetric cells (Figure 3, C and F and Figure S4B). These data confirm our hypothesis that a delay in mitosis induces Ace2 loss-of-asymmetry and it likely explains the Ace2 phenotype found in our primary screen with many of the class I cell cycle mutants (such as *mtw1*, *ipl1-2*, and *spc110-220*).

Ace2 asymmetric localization precedes cytokinesis and persists until G1 (Mazanka and Weiss 2010; Boettcher *et al.* 2012). Therefore, we tested whether there was a specific time when the loss-of-asymmetry caused by prolonged arrest was manifested. We imaged cells expressing Ace2-YFP, Nsg1-RFP (nuclear envelope), and Myo1-RFP (bud neck) after a controlled or prolonged arrest (Figure 3G and Figure S6). We quantified symmetric and asymmetric cells before and after cytokinesis, as determined by the disappearance of Myo1-RFP from the bud neck (Figure 3G and Figure S6) (Mazanka and Weiss 2010). Before cytokinesis, there was a significant increase in symmetric cells when subjected to the prolonged arrest (from 0 to 35% asymmetric cells, Fisher’s exact test *P* = 3 × 10^−14^, Figure 3H). In contrast, after cytokinesis the majority of cells were asymmetric with Ace2 only in the daughter nucleus (97% asymmetric cells, Figure 3J). We again found that symmetric cells had significantly higher levels of nuclear Ace2-YFP than asymmetric cells (Figure 3, I and K). These data show that Ace2 asymmetry is restored after cytokinesis. Taken together, our data show that prolonged mitosis caused, for example, by mutations in cell cycle regulator genes decreases Ace2 asymmetry and increases nuclear Ace2-YFP levels.

### The FACT complex is required for Ace2 asymmetry

**Figure 4:**
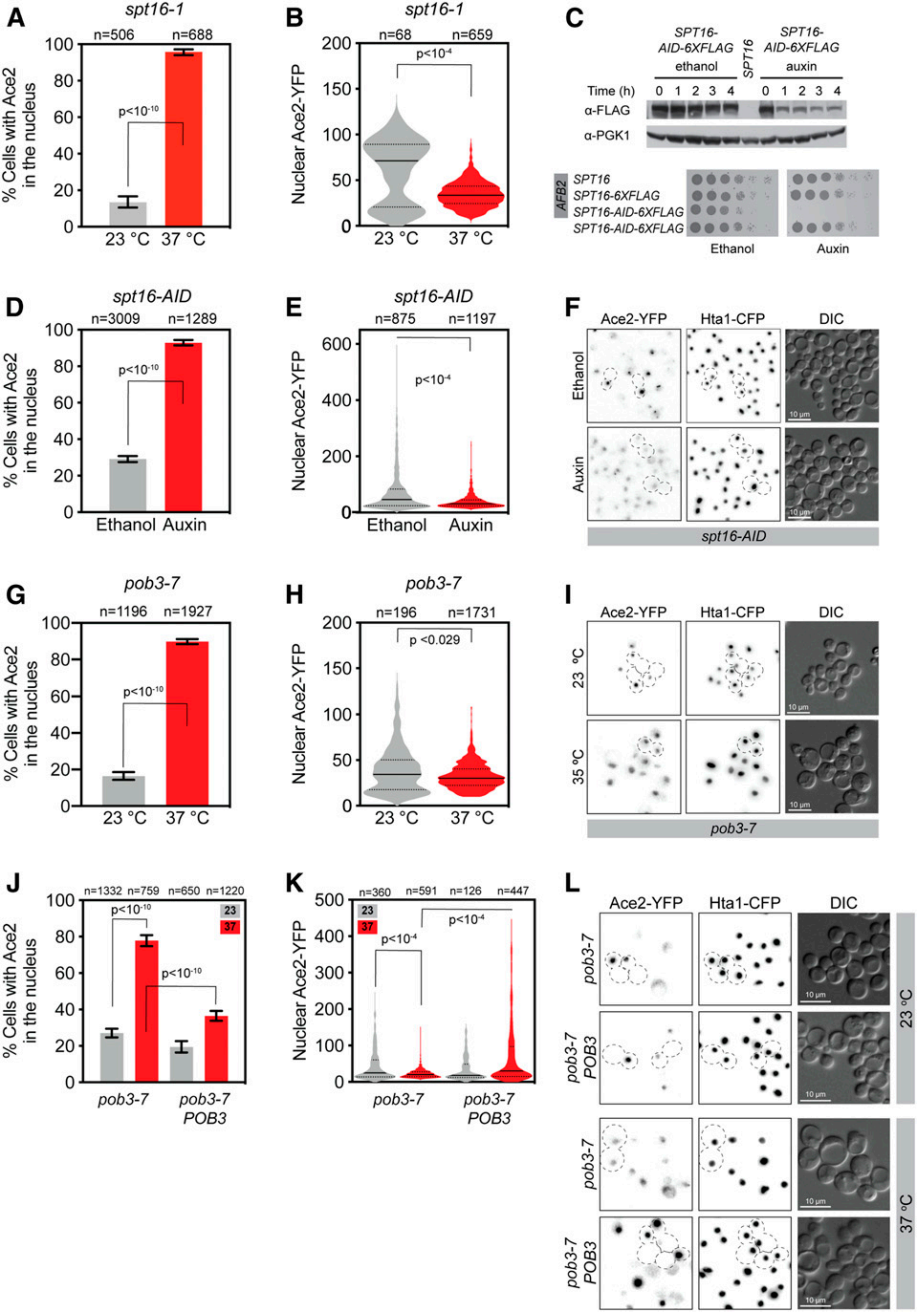
Class II mutants: the two members of the FACT complex Spt16 and Pob3 are required for Ace2 asymmetric localization. (A) Percentage of *spt16-1* cells with Ace2-YFP in the nucleus after growing for 5 hr at 23° or 37°. (B) Nuclear Ace2-YFP fluorescence intensity of *spt16-1* cells. The plot shows the frequency distribution of the values, the medians (solid lines), and the quartiles (dotted lines). Median (lower quartile 25%, upper quartile 75%): 23° 71 (21, 89), 37° 34 (25, 44). (C) Auxin-dependent degradation of *spt16-AID-6XFLAG* protein and spot test to assess cell viability. AFB2 is the E3 ligase that binds to AID. (D) Percentage of *spt16-AID* cells with Ace2-YFP in the nucleus when grown in ethanol (29%) or auxin (93%) for 5 hr. (E) Nuclear Ace2-YFP fluorescence intensity of *spt16-AID* cells. The plot shows the frequency distribution of the values, the medians (solid lines), and the quartiles (dotted lines). Median (lower quartile 25%, upper quartile 75%): ethanol 46 (25, 84), auxin 31 (24, 45). (F) Representative fluorescence microscopy images of *spt16-AID* cells grown for 5 hr in ethanol or auxin. (G) Percentage of *pob3-7* cells with Ace2-YFP in the nuclei grown for 5 hr at 23° or 37°. (H) Nuclear Ace2-YFP fluorescence intensity of *pob3-7* cells. The plot shows the frequency distribution of the values, the medians (solid lines), and the quartiles (dotted lines). Median (lower quartile 25%, upper quartile 75%): 23° 35 (18, 50), 37° 30 (23, 40). (I) Representative fluorescence microscopy images of *pob3-7* cells grown for 5 hr at 23° or 37°. (J) Percentage of *pob3-7* and *pob3-7 POB3* cells with Ace2-YFP in the nucleus: *pob3-7* at 23°, 27%; *pob3-7* at 37°, 78%; *pob3-7 POB3* at 23°, 19%; and *pob3-7 POB3* at 37°, 37%. (K) Nuclear Ace2-YFP fluorescence intensity of *pob3-7* and *pob3-7 POB3* cells. The plot shows the frequency distribution of the values, the medians (solid lines), and the quartiles (dotted lines). Median (lower quartile 25%, upper quartile 75%): *pob3-7* at 23°, 25 (14, 61); *pob3-7* at 37°, 20 (15, 28); *pob3-7 POB3* at 23°, 18 (13, 49); and *pob3-7 POB3* 37°, 30 (15, 97). (L) Representative fluorescence microscopy images of *pob3-7* mutant complementation. Error bars in (A, D, G, and J) correspond to 95% C.I.s. *P*-values were calculated with Fisher’s exact test (A, D, and G), the Wilcoxon rank sum test (B, E, and H), and the Kruskal–Wallis Test (K). CFP, cyan fluorescent protein; YFP, yellow fluorescent protein.

Only two mutant genes were identified that caused an abnormal presence of Ace2 in the nucleus of most cells at all cell cycle stages and abolished Ace2 asymmetry in telophase cells, *SPT16* and *POB3* (class II). We identified a single allele of *SPT16*, *spt16-1*, and two independent alleles of *POB3*, *pob3-7* and *pob3-L78R*. These two genes encode the two heterodimeric components of the FACT chromatin reorganizing complex (Formosa *et al.* 2001; Belotserkovskaya *et al.* 2003; Ransom *et al.* 2009; Xin *et al.* 2009; Hainer *et al.* 2012). When *spt16-1* cells were shifted to the restrictive temperature (Figure 2F), most cell nuclei contained Ace2 (13% at 23° *vs.* 96% at 37°, Figure 4A). We quantified the levels of Ace2 in the nuclei using fluorescence image analysis and found that Ace2 levels are typically lower than those of the daughter nuclei at the permissive temperature (Figure 4B). To confirm that these proteins are required for restricting Ace2 nuclear localization to daughter telophase cells, we created a degron allele of *SPT16* (*SPT16-AID*). This construct incorporates a C-terminal addition containing the target site for the AFB2 E3 ubiquitin ligase, whose interaction is dependent upon the presence of auxin (Nishimura *et al.* 2009; Morawska and Ulrich 2013). This allele was engineered at its endogenous locus. We found that the total levels of Spt16 were sharply reduced 1 hr after auxin addition and that cells were not viable in the long-term (Figure 4C). This strain allowed us to test whether we could recapitulate the abnormal presence of Ace2-YFP in most cell nuclei seen with the *spt16-1* strain. We incubated the cells with auxin (or ethanol as a control) for 5 hr and assessed the status of Ace2-YFP (Figure 4F). Similarly to *spt16-1* at the restrictive temperature, most cells grown with auxin contained Ace2 in the nucleus (29% with ethanol *vs.* 93% with auxin, Figure 4D) at levels that were consistent with those of *spt16-1* (Figure 4E).

Since both *spt16-1* and *spt16-AID* (Figure 4, A–F) are hybrids of the W303 and BY4741 genetic backgrounds, we confirmed the presence of nuclear Ace2-YFP in most cells in *spt16-1* cells isogenic for W303 (Figure S7, A and B). The percentage of cells with Ace2-YFP in the nucleus increased from 25% at 23° to 95% at 37° (Fisher’s exact test *P* < 0.0001). To confirm the presence of Ace2-YFP in the nucleus of cells at all cell cycle stages in the *spt16-1* mutant, we analyzed a subset of cells in G1 (round cells), S/M (budded cells with one nuclei), and telophase cells (separated mother and daughter nuclei). We found that all G1 and S/M cells contained Ace2-YFP in the nucleus at 37° (Figure S7C). The levels of nuclear Ace2-YFP were similar in G1, S/M, and telophase cells at the restrictive temperature, and telophase cells had reduced nuclear Ace2-YFP levels at 37° when compared with 23° (Figure S7E), indicating that the total levels of Ace2 are not increased in an *spt16* mutant. Moreover, all telophase cells at 37° were symmetric with all nuclei (both mother and daughter) containing Ace2-YFP (Figure S7C), and the AI index was sharply reduced to 0.14 ± 0.1 (n = 67, Table S3), indicating a strong loss-of-asymmetry caused by *spt16-1* mutation. Therefore, both the Ace2 asymmetry in telophase cells and the exclusion of Ace2 in the nucleus in G1 and S/M cells are compromised in *spt16* mutants.

Two *POB3* temperature-sensitive alleles, *pob3-7* and *pob3-L78R*, showed a sharp increase in the percentage of cells with Ace2-YFP in the nucleus at the restrictive temperature (Figure 4I and Figure S8). For *pob3-7*, the proportion of cells with Ace2 in the nucleus was increased from 16% at 23° to 90% at 37° (Figure 4G), consistent with the phenotypes of *spt16-1* (Figure 4A) and *spt16-AID* (Figure 4D). The amount of Ace2-YFP fluorescence in the nucleus was also decreased at the restrictive temperature (Figure 4H), although less than in *spt16-1* (Figure 4B), suggesting a less-severe phenotype. To confirm that the *pob3-7* mutation was responsible for the loss-of-asymmetry phenotype, we complemented the mutant strain with a wild-type *POB3* allele cloned into the *URA3* locus. We found that the complemented strain significantly decreased the proportion of cells with nuclear Ace2-YFP (Figure 4J) and restored the asymmetric localization of Ace2 in telophase cells at the restrictive temperature (Figure 4, K and L), thus confirming *pob3-7* mutation as the cause of Ace2 loss-of-asymmetry.

Since the *pob3-7* strain in Figure 4 was a hybrid of W303 and BY4741 genetic backgrounds, we confirmed abnormal Ace2 localization in *pob3-7* in W303 isogenic cells (Figure S9A). The proportion of cells with Ace2-YFP in the nucleus increased (24% at 23° to 70% at 37°, Figure S9B). When looking at different cell cycle stages, we found a sharp increase in cells with Ace2 in the nucleus from 23° to 37° in G1 (29–80%) and S/M phase (2–35%) in the *pob3-7* mutant. Moreover, the proportion of symmetric telophase cells also increased from 18 to 92% (Figure S9C). The insertion of a wild-type *POB3* at the *URA3* locus only partially complemented the *pob3-7* Ace2 phenotype in W303 isogenic cells, suggesting that the *pob3-7* allele may be partially dominant (Figure S9, B and C). The levels of nuclear Ace2-YFP were reduced in *pob3-7* mutant cells at the restrictive temperature (Figure S9D). However, in *pob3*-7 G1 cells Ace2-YFP nuclear levels increased at 37° (Figure S9E), similar to those found in *spt16-1* cells (Figure S7E). Consistent with only partial complementation, *pob3-7 POB3* cells also showed reduced nuclear Ace2-YFP levels (Figure S9D), but G1 cells had lower levels of nuclear Ace2 than *pob3-7* at 37° (Figure S9E).

### Spt16 is required for RAM network localization

**Figure 5:**
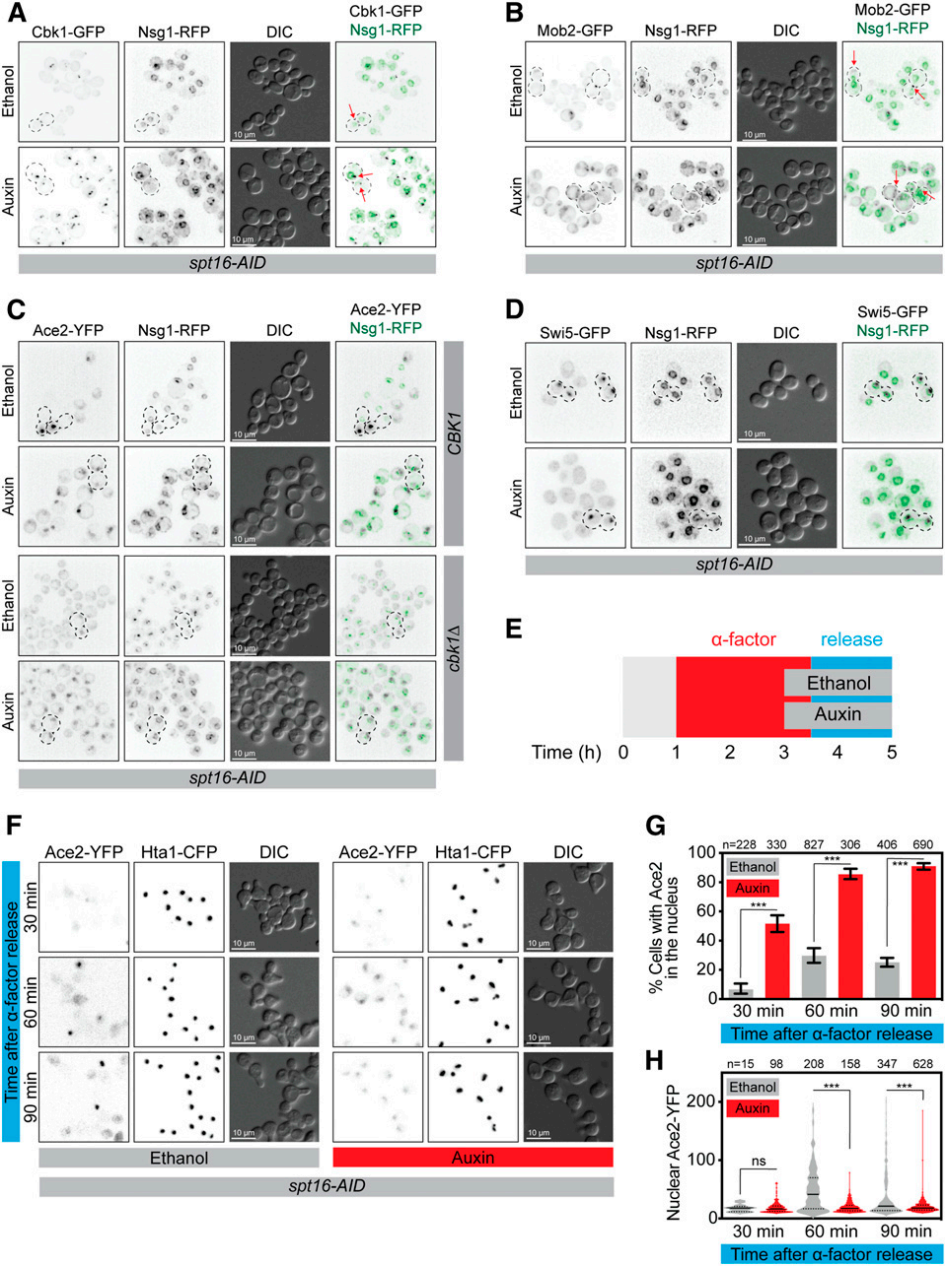
Cbk1-Mob2 complex is mislocalized upon Spt16 depletion. Fluorescence microscopy images of *spt16-AID* cells grown for 5 hr in ethanol or auxin. (A) Cbk1-GFP and (B) Mob2-GFP are mislocalized in Spt16-depleted cells. Red arrows depict GFP signal. These images were deconvolved for increased clarity. (C) Ace2-YFP localizes to most nuclei in Spt16-AID-depleted cells both in CBK1 (top panel) and *cbk1*Δ (bottom panel). (D) Swi5-GFP localization is not altered in *Spt16-AID*-depleted cells. (E) Experimental setup of G1 depletion of *Spt16-AID*. *SPT16-AID* cells were arrested in G1 with α-factor. Either ethanol (control) or auxin (*Spt16-AID* depletion) was added 1 hr before removing α-factor. Cells were imaged 30, 60, and 90 min after removal of α-factor. (F) Representative fluorescence microscopy of *spt16-AID* cells after G1 release. (G) Percentage of cells with Ace2-YFP in the nucleus. Error bars correspond to 95% C.I.s. *P*-values were calculated with Fisher’s exact test, ***P < 0.001. (H) Nuclear Ace2-YFP fluorescence intensity. The plot shows the frequency distribution of the values, the medians (solid lines), and the quartiles (dotted lines). Median (lower quartile 25%, upper quartile 75%): ethanol 30 min 18 (12, 22), 60 min 41(14, 70), and 90 min 21 (14, 50); auxin 30 min 17 (12, 22), 60 min 17 (13, 23, *n* = 347), and 90 min 18 (15, 23). *P*-values where calculate with the Kruskal–Wallis test, *** *P* < 0.001. CFP, cyan fluorescent protein; RFP, red fluorescent protein; YFP, yellow fluorescent protein.

The cell cycle-stage analysis of *spt16* and *pob3* mutants confirmed the complete misregulation of Ace2 localization: Ace2 loss-of-asymmetry in telophase cells and the abnormal presence of Ace2 in the nucleus in G1 and S/M cells. The RAM network mediates telophase asymmetric localization of Ace2 to the daughter nuclei by phosphorylation of the Ace2 NES by the RAM-Cbk1 kinase (Figure 1A). We examined the localization of Cbk1-GFP in strains depleted for Spt16-AID, also encoding the nuclear envelope marker Nsg1-RFP. Cbk1 is normally restricted to the bud tip in S phase and the bud neck during mitosis (Figure 5A and Figure S10). However, after depletion of *Spt16-AID* for 5 hr, we found that Cbk1 localization was profoundly disrupted with foci of Cbk1 often present outside of the nucleus (Figure 5A and Figure S10). We tested the localization of another component of the RAM network, Mob2. Mob2 is required for the specific localization of Cbk1 (Nelson *et al.* 2003). Like Cbk1, Mob2 was also mislocalized upon depletion of *Spt16-AID* (Figure 5B and Figure S11).

Since *SPT16-AID* *CBK1-GFP* and *SPT16-AID* *MOB2-GFP* strains are hybrids of the W303 and BY4741 genetic backgrounds, we confirmed the mislocalization of Cbk1 and Mob2 in *spt16-1* W303 isogenic cells (Figures S12 and S13). In *spt16-1* cells grown at 23°, Cbk1-GFP localized to the bud tip in budded cells and to the bud neck in mitotic cells (Figure S12). However, when grown at 37°, Cbk1-GFP localized to bright cytoplasmic foci (Figure S12). We then generated a *spt16-1 Mob2-RFP Ace2-YFP* W303 isogenic strain. When grown at 23°, Mob2-RFP localized to the bud neck in mitotic cells, and Ace2-YFP localized asymmetrically in the daughter nucleus in telophase cells (Figure S13). In contrast at 37°, Mob2-RFP bud neck localization was lost, and instead Mob2-RFP localized to cytoplasmic foci. As shown before, Ace2-YFP was localized in the nucleus of all cells. Moreover, the Mob2-RFP and Ace2-YFP signals did not colocalize (Figure S13). The Mob2-RFP signal at the bud tip in budded cells at both 23° and 37° in *spt16-1* cells was faint, possibly because of low fluorescence signals (Figure S13).

The mislocalization of both Cbk1 and Mob2 in *spt16-1* mutant cells suggests that symmetric localization of Ace2 in *spt16* mutants was caused by altered Cbk1 localization, which compromised its ability to phosphorylate Ace2 in telophase cells. To test this notion, we repeated the analysis of Ace2 localization with Spt16 depletion in a *cbk1*∆ mutant. If the mislocalization of Cbk1 in *spt16* mutant mediated Ace2 loss-of-asymmetry, we would expect no additional effect of *cbk1*∆ in Spt16-depleted cells. In the W303 genetic background *cbk1*∆ cells are viable (Bidlingmaier *et al.* 2001; Nelson *et al.* 2003). Therefore, we created Ace2-YFP *cbk1*∆ cells with the *SPT16-AID* allele. As previously reported (Mazanka *et al.* 2008), *cbk1*∆ cells showed Ace2 symmetry, but only in telophase cells (Figure 5C). *Spt16-AID* depletion in both wild-type and *cbk1*∆ cells led to Ace2 being present in most cell nuclei (Figure 5C), indicating that the effect of *cbk1*∆ on Ace2-YFP localization was epistatic to *Spt16-AID*. These data demonstrate that FACT contributes to Ace2 asymmetric localization through the localization of the RAM network.

### CDK phosphorylation is still active in spt16-depleted cells

Upon mitotic exit, Cdc14 must reverse CDK phosphorylation of the NLS to allow Ace2 into the nucleus (Archambault *et al.* 2004; Mazanka *et al.* 2008; Sbia *et al.* 2008) (Figure 1A). We hypothesized that CDK phosphorylation is perturbed in the FACT complex mutants, such that the NLS is always active (dephosphorylated). To test this hypothesis, we used the location of Swi5 as a surrogate. *SWI5* is a paralog of *ACE2* and encodes a transcription factor whose nuclear localization is also regulated by CDK phosphorylation and Cdc14 dephosphorylation of its NLS signal (Sbia *et al.* 2008). We reasoned that, if CDK phosphorylation of the Ace2 NLS is perturbed, then this would also be true for Swi5. We examined the localization of Swi5-GFP in a strain encoding a marker for the nuclear envelope (Nsg1-RFP) and found that depletion of *Spt16-AID* did not perturb Swi5-GFP nuclear recruitment (Figure 5D). We confirmed Swi5-GFP localization in *spt16-1* cells isogenic for W303. In *spt16-1* cells, Swi5-GFP was only visible in the nucleus of mitotic cells with separated nuclei, both at 23° and 37° (Figure S14). These data demonstrate that the upstream pathway of CDK phosphorylation is not disrupted in the FACT mutants. However, these data do not rule out a specific block to CDK phosphorylation of Ace2 itself.

The FACT complex may also interfere with nuclear export of Ace2. To test this notion, we attempted to suppress the *spt16-1* phenotype by overexpressing nuclear exportin *CRM1 (CRM1-OX*). Crm1 interacts with Ace2-NES and exports Ace2 from the nucleus (Jensen *et al.* 2000; Bourens *et al.* 2008). We transformed wild-type and *spt16-1* cells with a CEN (centromere) plasmid expressing *CRM1* from the *GAL1* promoter. We grew wild-type and *spt16-1* cells at 37° in either raffinose (control) or galactose (*CRM1-OX*) (Figure S15A). We found that *CRM1-OX* in wild-type and in *spt16-1* cells similarly decreased the number of cells with Ace2-YFP in the nucleus, from 51% to 35% and 93–83%, respectively (Figure S15B). *CRM1-OX* did not change the levels of nuclear Ace2-YFP (Figure S15C); these levels were significantly higher in wild-type that in *spt16-1* cells, as previously shown (Figure 4B). These data suggest that the nuclear export of Ace2-YFP is not affected in *spt16-1* cells.

### Spt16 is required for Ace2-YFP nuclear exclusion in G1

FACT mutants are inviable and *spt16-1* cells arrest in G1 at the restrictive temperature (Prendergast *et al.* 1990), suggesting an essential role of the FACT complex in G1. Ace2 is inactivated during G1 progression when Cbk1 phosphorylation decreases, leading to the export of Ace2 from the nucleus and its cytoplasmic retention (Mazanka and Weiss 2010). To test whether Spt16 plays a role in Ace2 inactivation during G1, we arrested *SPT16-AID* cells in G1 with α-factor and induced *spt16-AID* depletion by adding auxin 1 hr before releasing the G1 arrest (Figure 5E). Upon G1 release, control cells (growing in ethanol) progressed through the cell cycle and daughter-specific Ace2-YFP localization in telophase cells was visible after 60 min (Figure 5F, ethanol). In contrast, G1 arrest persisted in *spt16-AID*-depleted cells, as previously described (Figure 5F, auxin) (Prendergast *et al.* 1990; Morillo-Huesca *et al.* 2010). The proportion of cells with Ace2-YFP in the nucleus was higher in *Spt16-AID*-depleted cells (6.5% ethanol *vs.* 52% auxin) confirming a role of Spt16 in regulating Ace2 localization. In the control cells (ethanol), the proportion of cells with Ace2-YFP increased only to 25%, corresponding (Figure 5G) with telophase cells (Figure 5F) with high Ace2-YFP levels (Figure 5H). In contrast, in *Spt16-AID*-depleted cells (auxin), most contained nuclear Ace2-YFP (91% after 90 min; Figure 5G), and the levels of nuclear Ace2-YFP remained constant and low compared to control cells (Figure 5H). Taken together, our data demonstrate that the FACT complex is essential to maintain Ace2 asymmetric localization and cell cycle regulation. Although global Cdk1 phosphorylation is not altered in Spt16-depleted cells, RAM network Cbk1-Mob2 localization and G1 Ace2 cytoplasmic retention require the FACT complex.

## Discussion

We have systematically screened a collection of temperature-sensitive mutants of essential genes to identify those that perturb the asymmetry of a canonical marker of asymmetric cell fate determination, Ace2 (Figure 1). We found 81 genes whose disruption abrogates Ace2 asymmetric distribution and we grouped these into three phenotypic classes. The vast majority of mutants were class I, showing some but not all telophase cells with Ace2 mislocalized (Figure 2). This class includes many genes that are expected to disrupt the cell cycle, especially those involved in mitosis such as *MTW1*, *IPL1*, and *CDC20*. Other studies have shown that Ace2 asymmetry is perturbed in cells with altered nuclear morphology (Boettcher *et al.* 2012) and that asymmetric distribution of acentric DNA is altered by delayed mitosis (Gehlen *et al.* 2011). In line with these studies, we found that a prolonged mitotic arrest is sufficient to abrogate Ace2 asymmetry (Figure 3). These data, together with those described above, suggest that prior to the separation of the nuclei in dividing cells, Ace2 can diffuse between the two nascent nuclei, and that a rapid mitosis contributes to the generation of Ace2 asymmetry. We also found that Ace2 asymmetry was restored after cytokinesis, implying that the underlying mechanism (CDK phosphorylation and the RAM network) is functioning normally in these cells. We suggest that Ace2 present in the mother nucleus is exported due to the lack of Cbk1 phosphorylation that only occurs in the daughter (Mazanka *et al.* 2008). This suggests a mechanism that actively creates the asymmetric distribution of Ace2 (phosphorylation-dependent inactivation of NLS and NES signals) by balancing the diffusion of Ace2 along a concentration gradient. Interestingly, prolonged metaphase arrest increased the amount of nuclear Ace2, especially in symmetric cells, but also in the class I mutant *mtw1-1* (Figure 3). This increase in Ace2 protein level is presumably due to an increase of *ACE2* transcription, since *ACE2* expression is restricted to M phase (Spellman *et al.* 1998). Hence, it is possible that increasing the total amount of Ace2 protein contributes to the perturbation of its asymmetric partitioning.

In the class III mutants, asymmetric localization of Ace2-YFP in telophase cells was lost and Ace2-YFP localized to both mother and daughter cells. Within this class, we found mutants of components of the RAM network. The phenotypes of *mob2-14*, *mob2-28*, and *mob2-36* in our screen (Figure S3) were similar to the previously described *mob2*∆ mutant (Nelson *et al.* 2003). However, *mob2-38* and *hym1-15* (Figure S3) showed less-severe phenotype than *mob2*∆ and *hym1*∆ mutants, respectively (Nelson *et al.* 2003). Other RAM network components, such as Cbk1 and Kic1, were not represented in the temperature-sensitive collection. However, we found *cbk1*∆ to have a class III Ace2-YFP phenotype (Figure 5C). It would be interesting to compare the extent of Ace2 asymmetry in the RAM network kinase deletion mutants, such as *cbk1*∆ and *kic1*∆, with the conditional alleles studied here.

The class II mutants were limited to two genes, *SPT16* and *POB3*, which encode the two heterodimeric components of the FACT complex. The FACT complex plays an essential role in chromatin disassembly and reassembly during transcription elongation, and hence contributes to general chromatin structure maintenance (Reinberg and Sims 2006; Formosa 2008). In our study, *spt16* and *pob3* mutants abolished Ace2 asymmetry in telophase and, furthermore, contained Ace2 within the nuclei of cells outside mitosis (Figure 4 and Figure S7 and S9). The class II mutant Ace2 phenotype is similar to the constitutive nuclear accumulation found for Ace2-AAA/G128E, were the NLS cannot be inactivated by CDK (AAA) and the NES is inactivated (G128E) (Sbia *et al.* 2008), suggesting than the regulation of both nuclear import and export is compromised in FACT mutants.

The loss-of-asymmetry in telophase cells suggests that the phosphorylation of Ace2 by RAM-Cbk1 is globally affected in the class II mutants (Figure 4 and Figure S7 and S9). Indeed, we found that in *spt16* mutants, the RAM network components Cbk1 and Mob2 no longer localized to the bud neck during mitosis (Figure 5, A and B and Figure S10–S13). We have not ruled out the possibility that the changes in Cbk1 and Mob2 localization are caused by changes in the expression of *CBK1* and *MOB*2 in *spt16* mutants. Deletion of *CBK1* (*cbk1*Δ) was epistatic to depletion of Spt16 in terms of the Ace2 localization phenotype (Figure 5C). This is consistent with mislocalization of the RAM network contributing to the Ace2 loss-of-asymmetry of *spt16* mutant telophase cells.

We found Spt16 depletion in G1-arrested cells progressing into S phase and also nuclear accumulation of Ace2 in most cells (Figure 5, G–H). This suggests that Spt16 is required for Ace2 cytoplasmic retention in G1 (Mazanka and Weiss 2010). Both CDKs Pho85 and Cdc28 are required for Ace2 cytoplasmic retention in G1. Interestingly, the proportion of cells with Ace2 in the nucleus afte*r Spt16-AID* depletion was comparable to simultaneously mutating all Ace2 CDK phosphorylation sites (*ace2-AP* mutant) and deleting *pho85*∆ (Figure 5G) (Mazanka and Weiss 2010). This suggests that elevated levels of nuclear Ace2 seen in Spt16-depleted G1 cells may be the result of reduced CDK activity in G1. In *spt16* mutants, the downregulation of the G1 cyclins (*CLN1*, *CLN2*, and *CLN3*) leads to low CDK activity and defects in progression to START, the G1/S transition (Prendergast *et al.* 1990; Rowley *et al.* 1991). Therefore, it would be interesting to investigate the contribution of G1 cyclins to Ace2 cytoplasmic retention in G1 cells. Moreover, the presence of Ace2 in all cells, both mothers and daughters, suggests that Spt16 has additional roles in Ace2 cytoplasmic retention.

During mitosis, CDK phosphorylation of Ace2-NLS prevents Ace2 nuclear import (Dohrmann *et al.* 1992; Archambault *et al.* 2004; Mazanka *et al.* 2008; Sbia *et al.* 2008). Hence, low CDK activity in *spt16* mutant cells could explain the presence of Ace2 in S/M cells (Figure S7). However, we found that the cell cycle regulation of Swi5 nuclear localization was not altered in *spt16*-depleted cells (Figure 5D and Figure S14), suggesting that CDK-mediated NLS phosphorylation in mitosis was not primarily affected.

Taken together, we found that essential processes affect the asymmetric distribution of Ace2 protein. A mitotic delay reduced Ace2 asymmetry but did not compromise Ace2 cell cycle regulation. Diffusion of proteins from one nascent daughter cell to the other is likely prevented by rapid mitosis and cytokinesis. Based on previous studies (Gehlen *et al.* 2011; Boettcher *et al.* 2012), we suggest that mitotic delay allows diffusion to break the asymmetry of many different asymmetrically distributed molecules. We found a novel, critical role of the FACT complex in maintaining the correct localization of both Ace2 and the RAM network. The FACT complex also affects the nuclear levels of Ace2 in G1 cells. It will be relevant to determine the mechanism by which chromatin reorganizing is involved in the localization of the RAM network proteins. Furthermore, it will be of interest to investigate whether chromatin-reorganizing factors, such as the FACT complex, play a role in the localization of conserved *hippo/ndr* kinases in other eukaryotes.
